# Silicon-Based Sensors for Biomedical Applications: A Review

**DOI:** 10.3390/s19132908

**Published:** 2019-07-01

**Authors:** Yongzhao Xu, Xiduo Hu, Sudip Kundu, Anindya Nag, Nasrin Afsarimanesh, Samta Sapra, Subhas Chandra Mukhopadhyay, Tao Han

**Affiliations:** 1 School of Electronic Engineering, Dongguan University of Technology, Dongguan 523808, China; 2 CSIR-Central Mechanical Engineering Research Institute, Durgapur, West Bengal 713209, India; 3 DGUT-CNAM Institute, Dongguan University of Technology, Dongguan 523106, China; 4 School of Engineering, Macquarie University, Sydney 2109, Australia

**Keywords:** silicon, sensors, biomedical, semiconducting, nanowire

## Abstract

The paper highlights some of the significant works done in the field of medical and biomedical sensing using silicon-based technology. The use of silicon sensors is one of the pivotal and prolonged techniques employed in a range of healthcare, industrial and environmental applications by virtue of its distinct advantages over other counterparts in Microelectromechanical systems (MEMS) technology. Among them, the sensors for biomedical applications are one of the most significant ones, which not only assist in improving the quality of human life but also help in the field of microfabrication by imparting knowledge about how to develop enhanced multifunctional sensing prototypes. The paper emphasises the use of silicon, in different forms, to fabricate electrodes and substrates for the sensors that are to be used for biomedical sensing. The electrical conductivity and the mechanical flexibility of silicon vary to a large extent depending on its use in developing prototypes. The article also explains some of the bottlenecks that need to be dealt with in the current scenario, along with some possible remedies. Finally, a brief market survey is given to estimate a probable increase in the usage of silicon in developing a variety of biomedical prototypes in the upcoming years.

## 1. Introduction

One of the cornerstones in the field of electronics has been the employment of sensors for the ubiquitous monitoring of different applications. Among the different raw materials being processed by researchers to fabricate the sensors, MEMS-based sensors [1,2] have been the most important. The earliest form of MEMS sensors, which were used to monitor day-to-day applications, dates back to the late 80s and early 90s [3]. With time, the concept of Microelectromechanical Systems (MEMS) has developed in a much easier manner to create a miniaturised version of the sensing prototypes using the microfabrication technique. The usage of MEMS-based sensors has, with time, broadened to a wide range of applications [4,5,6], including different kinds of alloys and pure material [7,8,9]. Among the different kinds of MEMS-based sensors, silicon sensors have always been crucial [10,11] for quick and efficient sensing purposes. In comparison to other MEMS-based sensors, the advantages of the silicon sensors involve their small size, excellent signal-to-noise ratio, low hysteresis, ability to work in extreme environmental conditions, and high repeatability in their fabrication structure and responses. One of the significant characteristics of the silicon sensors is their higher response to the changes in frequency in comparison to their enlarged counterparts. These sensors are semiconducting where the substrates are developed from single-crystal silicon. These single-crystal silicon sensors have been developed, characterised and employed to a large extent depending on their type and working principle. The advantages of semiconducting prototypes for sensing purposes lie in the simplicity of their structure and working principle, dynamic nature suitable for implementation, low cost, and scalability. The fabrication of these silicon wafers involves nine stages before they are sent for the fabrication of sensing prototypes—these nine stages are: ingots, peripheral grinding, slicing, beveling, lapping, etching, heat treatment, polishing, and cleaning [12]. The silicon sensors have been implemented in a wide range of applications: ranging from personalised use like healthcare to industrial uses. Among all the applications, the use of silicon sensors for biomedical application has been one of the major sectors where they have, for a while, been creating a major impact [13,14,15]. The potentiality of these sensors has hence been increased by integrating them with embedded electronics to serve multifunctional medical applications. These silicon sensors are processed by sophisticated micromachining processes to form substrate-like material to be subsequently processed for specific applications. One of the common techniques for processing silicon substrates is to undergo photolithography [16,17] to form the electrode designs on the substrates. This photolithographic technique is further combined with other methods, like etching, thermal oxidation, spin-coating, and sputtering, to develop the sensing prototypes. Some of the arguments in favour of using silicon sensors for biomedical sensing comply with their miniaturised size, high reliability, quick response, lightweight nature, biocompatibility, and minimal hysteresis in their responses. Among the wide sector of biomedical sensing applications that the silicon sensors have been employed for, some of them are applied to physiological body movements [18], human bio-signals like heartbeats [19], blood flow [20], pulse rate [21], drug delivery [22], protein [23] and tumour detection [24], DNA sensing [25], and stem cell research [26]. The performance of the sensors, however, depends on the efficiency in terms of the sensitivity, resistance toward the change in temperature and humidity, robustness, and the enduring nature of these. The biggest advantage, in terms of the connection and trans-reception of data, is the ability of the silicon sensors to be easily attached to the entire signal conditioning circuitry to form the integrated circuitry (IC). Table 1 shows a comparative study between some of the silicon sensors used in different biomedical sensors, along with their strengths and drawbacks. Different kinds of silicon sensors have been used for medical sensing. Some of them are based on piezoelectricity, piezoresistivity, electromagnetic and capacitive sensors principles. The working principle of the sensors depends on the applications for which the sensors are being used.

The researchers have been trying to focus on the adoption of the silicon-based sensing systems for ubiquitous monitoring and to possibly determine the anomaly related to some of the health problems. Although a lot of work review papers have been written [27,28,29,30] on silicon-based sensing, there are still some loopholes with the review process that need to be addressed. First, none of the papers has especially addressed an exclusive use of MEMS-based sensors for biomedical sensing. All these papers have considered more than one application, without further elaborating on every aspect. Some of the papers, like [31], have only detailed the work on similar applications of biomedical sensing, like the use of microsurgical tools and microfluidic sensors. Second, among the different types of MEMS sensors, the applicability of silicon sensors, specifically to sense different parameters, has not been described yet. Third, even though some papers [32] have partially worked on the MEMS and silicon-based technology, explaining some of the wireless sensing systems, they have primarily focused on the communication part of the integrated system, rather than on the sensing operation. Some papers have considered the explanation of the significance of silicon-based sensors for certain diseases [33,34], without explaining the need for sensors. Consequently, it is state-of-the-art to elucidate a review paper highlighting some of the significant research work done on silicon-based sensors, which have been particularly used for medical sensors. The use of silicon sensors has to be explained in terms of their fabrication and implementation for a certain aspect of the health parameter. This paper highlights some of the significant work that involves the silicon-based sensors for monitoring biomedical applications. The novelty of this work lies in the explanation of some of the significant work done by the silicon sensors for a wide range of biomedical sensing parameters.

The manuscript has been sub-divided into four sections. The introduction given in the first section showcasing the importance of silicon-based sensors for biomedical sensing is followed by Section two, which explains some of the works done on the detection of health parameters using silicon sensors and the corresponding embedded systems. This section presents seven different types of work, based on the nature of the use of silicon for biomedical sensing. The third section elaborates about the challenges related to some of the existing sensors, as well as some of the possible remedies that can be associated with them. It also provides a glimpse into future opportunities in terms of market surveys on silicon-based sensors. The conclusion of the paper is presented in the final section of the manuscript.

## 2. Utilisation of Silicon for Biomedical Applications

The proposition for the use of silicon for medical applications has existed for almost four decades now [42,43]. Since then, silicon has been used in different forms for a range of in-vitro and in-vivo applications. The different forms of silicon have been mechanically and electrically enhanced via microfabrication technologies. For the last three decades, silicon-based sensors have found significant applications in industry and medicine [44,45,46]. The origin of silicon-based sensors can be found in 1954, when Smith et al. for the first time introduced the term “piezoresistivity” by studying the stress sensitive effects in silicon along with germanium [47]. During the early 1960s, the first silicon pressure sensors and strain gauges were developed and reported from the Bell labs and the Honeywell Research Centre [48]. During these years, silicon sensor technologies had become quite popular, and by the late 1960s, different US companies had already produced the first silicon pressure sensors. Furthermore, the combination of silicon technology with information and communication technologies enabled the development of compact, low-cost and high-performance devices for different applications [49,50,51,52]. Nowadays, biological and biomedical silicon-based technology has exhibited a remarkable potential in the application field, from a research point of view as well as an industrial perspective [53,54].

### 2.1. Planar Sensors

As to the use of silicon-based prototypes as planar sensors, researchers have used them for electrochemical sensing for biomedical applications [55]. Due to their structural advantages, like the variation of the distance between the two electrodes in order to simultaneously vary their electrical characteristics, planar sensors possess certain advantages like a lower input power, higher efficiency and robustness, and higher ionic diffusion. Consequently, these sensors are highly efficient for developing a lab-on-a-chip system for biomedical applications. Due to their reduced scanning area for the nanoscaled electrodes, the sensitivity is higher when compared to other electrode patterns. In planar capacitive sensors, the sensor electrodes are positioned in a coplanar surface. In addition to the mentioned features, the planar configuration offers the possibility of evaluating a material under test (MUT) from one side only [56], which is especially useful when the access to MUT is restricted. These characteristics make capacitive sensors a vital device for applications, such as non-destructive testing (NDT) [57], proximity and displacement measurement [58,59], material characterisation [60], and imaging [61,62]. Planar capacitive sensors operate based on parallel-plate capacitors. The electric field lines bulge from one to another due to the planar nature of the sensors. This leads to the formation of fringing electric fields. When the electrodes open to a coplanar plane, the fringing electric field becomes the largest between the working and sensing electrodes [63]. The fabrication of the sensors required a thin-film technology, along with photolithography and etching techniques to form the respective substrates and electrodes. Some of the substrates used to form the sensors were silicon nitrate, silicon dioxide, polycrystalline silicon and aluminium. The thin-films were formed using the lift-off technique through a stencil, followed by the evaporation process to deposit the metal on the wafer. Then, the stripping process was carried out for the operation metal to obtain the contacts of the sensors at defined patterns. Finally, sputtering and vacuum evaporation was carried out to define the electrodes of the sensors. The electrodes were developed in the form of interdigital capacitive sensors for certain advantages, like a small voltage drop, small charging currents, and a fast steady-state mass transfer. The dimension of the microelectrodes was less than 20 µm, where the space between two consecutive electrodes is in single micro units. The thickness of the metallic layers on these interdigitated electrodes was around 250 nm for polycrystalline and monocrystalline silicon. The detection of urea, DNA and other proteins was done using these sensors via conductimetric measurements. Musayav et al. [64] developed a microarray sensor for detecting the direct phosphate backbone charge of DNA molecules. Capacitive metallic electrodes with dimensions of 7 μm × 7 μm had been utilised to develop the array by arranging the electrode with a pitch of 15 μm. Since the sensor was used for a low-noise sensing capability and amplification circuits, along with double sampling, the sensitivity has been quite improved, and it was found capable of sensing DNA having a 1 pM concentration. This research group also noticed that surface treatment is required to improve the sensitivity, and, therefore, the gold coating was retained for future work. Capacitive sensors have been employed in some applications because of their low cost, quick response, as well as their non-invasive and no radiation features in electrode design [63,65].

Afsarimanesh et al. [66] have also used silicon-based planar sensors to determine the change in concentrations of CTX-1, which is one of the biomarkers used to study the condition of the bone. The change in the concentration of CTX-1, which is one of the fundamental proteins, has been studied by the researchers to determine the amount of resorption of bone. Specific concentrations of CTX-1 ranging between 0.1 to 2.5 ppb were produced using a serial dilution to determine the capability of the silicon sensors to measure them. This range of concentration was decided based on the amount of CTX-1 being released from a patient who had osteoporosis. Electrochemical Impedance Spectroscopy (EIS) was used to study the response of the sensors in terms of the change in impedance with respect to the frequency. The silicon sensors fabricated with the photolithographic technique were coated with a polymeric layer, which consisted of templates specific to the CTX-1 molecule. The electrodes were interdigital, operating on the planar capacitive principle. The sensing area of the sensors was 2.5 mm × 2.5 mm. The molecularly imprinted polymer was formed to coat a colloidal layer on top of the sensing surface to make the sensor sensitive toward the particle CTX-1 molecule. The sensitivity of the sensors was high, with a quick response of less than one second. The sensor was integrated with a microcontroller-based system to make it portable for real-time applications. The development of the embedded system signifies some other qualities of the sensors. Some of these include the quick data-collection process and testing of samples from different patients at a very short time. Other advantages of this system are its low-cost, ease of operation, robustness, and a very low detection limit.

### 2.2. Polysilicon-Based Sensors

The next category is the use of polysilicon to develop the sensing parts of the prototypes. The advantages of using polysilicon lie in the controllability of the thickness of the formed prototypes, their durability within high changes in temperature, ability to tune in the resistance to simultaneously change the temperature coefficient of the resistance, and compatibility to form hybridised prototypes in conjugation with other metals. Fernandez et al. reported a triglyceride measurement system [67], which was made of composite porous silicon/polysilicon micro-cantilevers. Micro-cantilevers can transduce different types of chemical and physical occurrences into mechanical motion. Micro-cantilevers have been used in a range of applications, such as those required for sensing cells, proteins, and metals. Arrays of these cantilevers can detect multiple factors at once. This biosensor has a cantilever beam with a length of 100–200 µm, width of 10–20 µm, and a thickness of 2 µm. Abdelghani et al. [68] fabricated a silicon-based capacitive pressure sensor to measure the blood pressure and heart rate by studying the deflection of the diaphragm resulting from the applied pressure. The pressure causes deformation on the movable upper plate and creates a gap between the upper and the fixed lower plate, which generates the capacitance. This research group also established the FEM model to analyse the deformation of the membranes. Due to its low power consumption and advantages resulting from miniaturisation, it has a great capacitive response within the pressure range of 0–40 kPa.

### 2.3. D printed and Optical Sensors

**Figure 1:**
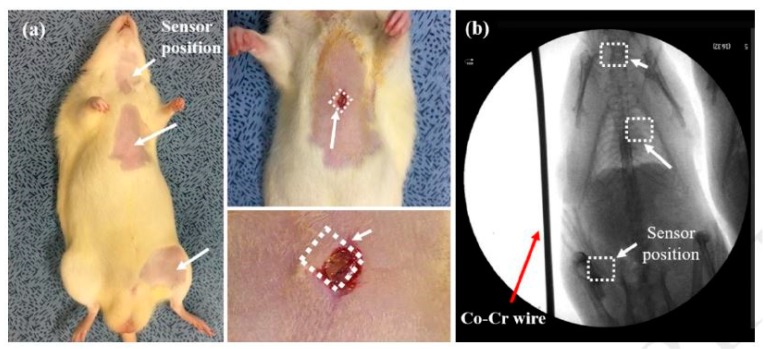
The insertion of wireless pressure sensors into the animal was done (**a**) at three different places (**b**) to perform the biocompatibility test and determine the blood pressure [69]. The image has been reproduced with permission from [69].

With the advancement in the field of 3D printing, researchers have begun to emphasise the development of 3D printed silicon-based sensors for biomedical applications. These sensors are quick to fabricate, able to be customised in a wide range, robust in nature and highly durable. One of the significant issues with these materials is their biocompatibility, especially for the sensors that are used for in-vivo applications. The significance of these 3D sensors lies in their integration with other organic and metallic elements to form sensing prototypes [69]. These sensing systems have an additional attribute of transmitting the monitored data wirelessly for smart health monitoring. Other characteristics of these sensors are their biodegradable nature and their stability in their responses for over three months. The sensors are operated as battery-less capacitive and inductive pressure sensing prototypes at high resonating frequencies of 130 and 183 MHz. The sensors have been used to measure the change in blood pressure of different animals. The fabrication process started with the deposition of a copper layer of around 200 nm on top of the 300 nm thick oxidised silicon layer. Electroplating was done on the copper layer to form moulds to define the inductor coils. This was followed by photolithography to form the patterns on the substrates. Then, the SU-8 layer was used to form the topmost part of the sensor with a thickness of 100 µm. Then, the oxidised silicon layer below the SU-8 layer was removed by a buffer solution, followed by forming a bottom layer of SU-8. Finally, electrodes were formed with Ti and Au with a thickness of 10 nm and 100 nm, respectively. The sensitivity of the measured pressure range for these sensors was around 160 kHz/mmHg, with an error range of 2%. The sensors were found capable of determining the change in phase angles with respect to a frequency range between 100–180 MHz for different movements of the attached antenna. The validity of the biocompatibility and the experimental results from these sensors were obtained by inserting these sensors in three different locations in the body of a mouse, as shown in Figure 1 [69]. The sensors could successfully measure the blood pressure of the animal, ranging between 91–129 mmHg. The robustness and repeatability in the responses were also validated by shifting the resonating frequency within a range of 3 to 7 MHz.

Initially, the use of silicon in terms of optical sensors was done by developing a fibre-optic pressure sensor [70] for biomedical applications. In addition to their small size, their resistance toward electromagnetic radiation and their ability to monitor at remote distances make them a popular choice for biomedical applications. The dimensions of the sensors were 270 µm × 270 µm × 150 µm. The working of the sensors is based on the principle of detection of the intensity of reflection of light from a diaphragm. The biomedical use of these sensors is based on balloon catheters. The catheters were formed with Polyethylene terephthalate (PET) and had a diameter of 1.5 mm. Anisotropic etching and direct wafer bonding techniques were used to fabricate the sensor. The sensing structure consisted of Gold and Chromium thin-films, along with a silicon-based optical fibre-stopper and fibre-aligned structures. The fibre-stopper was formed using a sodium silicate solution, whereas the alignment was done between the bottom and middle silicon wafers by pressing with a rubber roller. The sensors were annealed at 200 °C for two hours to achieve a proper attachment and alignment between the two layers of the sensors. The incident light was from an LED having a wavelength of 1.31 µm. The optimisation was done on the area and thickness of the working environment to operate on a pressure range of 0.1–1 MPa. The response of these sensors to such a high range of pressure also increases their significance for a wide sector of applications operating at temperatures at middle and high ranges. The pressure was achieved using a dry nitrogen chamber to determine the intensity of the reflection with respect to the variation of the pressure. The sensors obtained an average responsiveness of 1.9 µmW MPa^−1^.

### 2.4. Ion-Sensitive Field-Effect Transistors

Sensors were also developed in the form of Field-Effect Transistors (FETs) and Ion-sensitive Field-Effect Transistors (ISFETs), where the gate is made sensitive to particular ions, such as Na^+^, NH_4_^+^, K^+^ and others [71,72]. These types of sensors have gained a significant importance due to their employability in biomedical applications, which results from their ability to measure different kinds of analytes, swiftness of response, high robustness, and low output impedance. The techniques to fabricate FETs and ISFETs have been consistent over the years, with their resultant sizes decreasing every day to increase their efficiency and sensitivity. This performance can also vary commensurate to the level of doping done on every individual sensor. The sensors were also used for bio-sensing to measure certain elements, such as a2–interferon [73] and β–Bungarotoxin [74]. Here, the sensors were able to detect the toxin present in nano levels. One of the earliest works related to the use of silicon for biomedical sensing has been on the employment of silicon needles [75] to develop ion-sensitive field effect transistors (ISFET).

The sensors consisted of multi-sensing silicon needles, including a pseudo-reference electrode developed from platinum and a temperature sensor. The electrode and temperature sensor were developed via CMOS-compatible technology and a silicon micromachining technique. The sensors displayed a high sensitivity and good linearity toward the detection of myocardial ischemia during cardiac surgery. The applications in cardiac surgery with these sensors were determined by the change in pH and pK. P-type silicon wafers were used with a resistivity of 4–40 Ω cm, having a high doping level of 1 × 10^15^ cm^−3^. The ISFETs were fabricated via a photolithography technique, at six different levels, having a gate and LPCVD of silicon dioxide and silicon nitride, respectively. The shape of the silicon needles was defined through a reactive ion etching process, which was independent of the crystalline planes of the silicon surface. The length and width of the needle were around 7 mm and 0.8 mm, respectively. The sensors were equipped with a membrane of plasticised potassium, which makes them highly sensitive towards potassium. The sensors showed a change toward the pH and temperature, obtaining a range of linear responses between 8 × 10^−5^ and 8 × 10^−2^ M and a sensitivity of 50 ± 2 mV per electrode. The response time and temperature coefficient of the resistance of the ISFETs were one second and 2687 ppm/C.

### 2.5. Silicon-On-Insulator Sensors

Another work based on silicon-based sensors for biomedical sensing from a different sector was the employment of silicon as insulators to form wireless implantable sensors [76]. These sensors were used for fully implantable cochlear prosthesis and, brain sensing technology, consisting of an embedded signal-processing circuit. These systems hold a key role for the human prosthetic and brain-machine interfacing systems. The system was fabricated based on a silicon-on-insulator (SOI) technique to design the proposed architecture. Some of the advantages of this system include the simple design, common-mode interference rejection, low noise, null DC power dissipation, and straight forward interfacing between the sensor and the system. One of the matters under consideration was that the acceleration noise floor limited the resolution of the device. This phenomenon was due to the Brownian noise, which was directly proportional to the resonant frequency of the mechanical device, and inversely proportional to the mass and quality factor. The accelerometer consisted of a set of sensing fingers having a thickness of 25 µm, width of 2 µm and an overlap dimension of 96 µm. The sensing surface of the system was around one mm^2^, while the entire system had a dimension of 2.5 mm × 6.2 mm, obtaining a capacitance of 2 pF. The sensitivity and the resonant frequency obtained by the system were 11.5 mV/g and 6.4 kHz. For the cochlear prosthesis measurements, the system was able to detect 60 dB SPL, 35 dB SPL and 57 dB SPL at 500 Hz, 2 kHz and 8 kHz, respectively. For the brain-sensing technology, the sensors were able to distinguish between the on and off states for a spectrogram low-pass filter recording of a person with Parkinson’s disease. The measurements were taken within the chosen beta band between 10 and 20 Hz. Local field potential signals were recorded at low frequencies of around 200 Hz to reduce the generated noise. The Chopper-stabilization mode was employed to determine the noise performance for the operation of the system, in order to improve the common-mode rejection ratio. The stability and the biocompatibility of the sensing system were ensured by the size and type of the chosen materials. Commercially available macro electrodes, developed from an alloy having an impedance of around one kΩ, were chosen to form the conductive parts of the sensors. The response time of the sensors to detect interictal epileptiform singles was more than one second. Even though the developed sensors were operated over a wide range of frequency, one of the disadvantages of these types of sensors was found to be their high output impedance, which increases the power required to drive the system. The sensors were also not as small as the other types, which eventually decreased their sensitivity. One of the other points on which the researchers can focus in this work is the reduction of Brownian noise generated in the system. Other works on Silicon-on-Insulator wafers were conducted using the coating on PZT thin films while fabricating standard piezoelectric MEMS microfabrication technology that generated ultra-thin PZT/Si structures [77,78].

### 2.6. Silicon Nanowires

**Figure 2:**
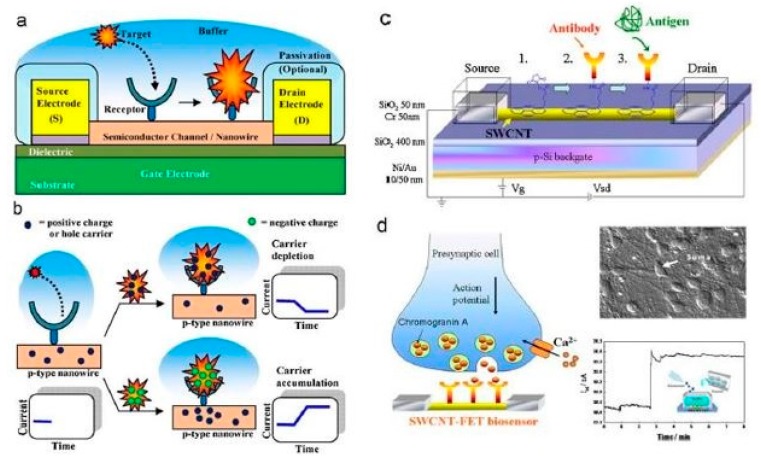
(**a**) Schematic diagram of the nanoscaled-FET silicon-based sensors. (**b**) Binding of the target molecules with the receptors. (**c**) Surface modification of the FET device done with a π-π interaction process, immobilisation and detection. (**d**) Release of the neurons on the sensing surface of the FETs to determine the change in current with respect to the time [81]. The image has been reproduced with permission from [81].

There has been a tremendous increase in the use of nanowires because of their significant advantages, such as a high efficiency, high selectivity and sensitivity. Silicon nanowires have been developed along the same lines, keeping in mind the aforementioned advantages, and hence served the same purpose. Silicon nanowires hold a huge potential as conductive materials due to their widely accepted attributes. Some of them include a high electrostatic controllability [79] with better current characteristics and a lower noise density. Furthermore, in comparison to metallic nanowires like iron, silicon nanowires are relatively easier to fabricate. This is due to the low-pressure operation, the feasibility of the fabrication of these nanowires in batches and the low-cost setup. Another advantage of these sensors is their good orientation, unlike certain nanoparticles like carbon nanotubes, where the orientation is effected by the tunneling current that exists between them [80]. Silicon nanowires have shown the potential to be used for both electrochemical and pressure sensing applications in the field of biomedical sensing. The range of analyte being detected with the help of these nanowires is also exceptional. Chen et al. [81] explained the use of silicon nanowires in developing field-effect transistors for the detection of proteins, DNA sequences, small molecules, and other biomarkers for certain diseases like cancer. The physiological responses of the SiNWs-FETs from the cells and tissues were also measured. The fabrication and experimental processes are shown in Figure 2 [81]. The sensing system consisted of three-electrodes, namely the source, drain, and gate electrodes, which formed a bridge on the semiconducting channel. The channel was developed from SiNW, which was responsible for the attachment of bio-receptors via a chemical modification. The attachment of the receptors was completed to recognise the specific target analyte, in order to achieve a high sensitivity, specificity and strong affinity. A detection system was used to determine the variation in the conductance and surface potential of the channel during the interaction between the receptor and target. Two different techniques, namely top-down and bottom-up, were used to fabricate the silicon nanowires on oxidised silicon wafers with a thickness of around 200–400 nm. The nanowires and the connecting electrodes of the SiNW-FETs were formed on the sensors using a range of techniques like photolithography, ion-implantation, reactive ion etching (RIE), electron-beam lithography, and thermal evaporation. With this approach, the width of the nanowires was around 100 nm. The silicon substrates were doped with boron while the nanowires were doped with different elements like nitrogen and phosphorous to alter their semiconducting property. The bottom-up approach was done through a chemical vapour deposition process, which was followed by the assembly of the formed nanowires, using an electron-beam lithographic process. The advantage of this process is associated with metallic nanoparticles having controlled sizes with the help of a catalytic activity. Then, the nanowires were assembled using techniques like flow-assisted alignment, the Langmuir-Blodgett process, being bubble-blown, smearing-transfer, roll-printing, and PDMS-transfer. After the dispersion of the nanowires on the oxidised silicon wafers in the central areas to form the channels, they were subsequently connected with metallic electrodes. The electrodes were then coated with an insulating layer to minimise the leakage of the current during the experiments. The sensor consisted of a sensing area of around 1.5 mm × 1.5 mm containing the FET array, a microfluidic channel and a detection system. The sensing system was finally mounted on a plastic circuit board having electrical connections built with aluminium wires of a diameter of 30 µm. During the experimental process, the FETs were immersed in acidic buffer solutions, utilising the high surface-to-volume aspect ratio of the nanowires. This affected the external electric field, which subsequently changed the resultant conductance of the devices. Some of the biomedical experiments carried out with these devices were protein-protein interactions, DNA hybridisation, the monitoring of viral infection, peptide-small molecule interactions, and biomarker detection. The corrosion of the source and drain electrodes was prevented during the experimental process in harsh environments. The FET-probe of the sensors had a sensitivity of 4–8 µS/V for the tested solutions.

**Figure 3:**
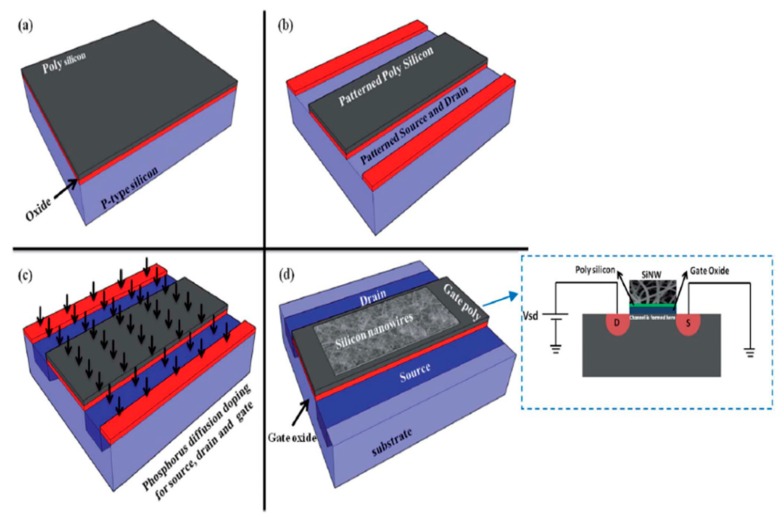
The schematic representation of the skein SiNW incorporated FET fabrication process. [82]. The image has been reproduced with permission from [82].

Another use of silicon nanowires has been shown by Abdolahad et al. [82] by developing a biosensor for the electrical charge sensing of cell membranes. Silicon nanowires, with the help of a gold catalyst layer, were fabricated in a low-pressure chemical vapour deposition (LPCVD) system for the electrical monitoring of the negative charges that are present on the membrane. It has been observed that during the detection of the negative particles on the surface of the cancer cell membrane, the primary colon cancer discharges more current, confirming progressive colon cancer. Thus, by differentiating between the electrical responses received from the device according to the different negative charge productions, cancer cells can be differentiated or categorised. The silicon object is coated with a 10 nm gold thin catalyst layer on which the specimen needs to be positioned inside the LPCVD system. This SiNW bioFET can be very useful as a diagnostic tool for cancer examination in future biomedical applications. Figure 3 depicts the schematic diagram of the FET fabrication process [82]. Another similar study had been done by Zhang et al. [83] for DNA sensing using field-effect based silicon nanowire (SiNW) sensors. Since the gap between the DNA charge layer and the SiNW surface is highly dependent on the sensitivity, in terms of the ionic strength of the electrolyte solution, it becomes weaker when the gap is further increased. This depends on the hybridisation spots of DNAs to the peptide nucleic acid (PNA) capture probes. Polysilicon layers had been covered on the thermally grown oxide on a p-type test wafer in order to design this biosensor with a dimension of a few tens of a nanometre.

Carrara et al. have been working for a while on the fabrication of memristors with silicon nanowires [84] and their further implementation for biosensing purposes [85,86]. A combination of the Complementary Metal Oxide Semiconductor (CMOS) processing technique and photolithography was used to develop poly-crystalline silicon nanowires that exhibited memristive behaviour. After developing an oxidised layer of silicon on the silicon substrate, a thin film of polysilicon was developed by LPCVD and deposited with a thickness ranging between 40 and 90 nm. This was followed by the etching process to remove the horizontal layer. A spacer was defined, which was reminiscent of the nanowires. The nanowires were polysilicon in nature, having thicknesses from 20 to 60 nm. The contact regions of the nanowires were developed with chromium and nichrome. These devices displayed an ambipolar behaviour with an increase in the conduction of the current due to the presence of holes. For the computational study, their behaviour was studied under a certain frequency range to determine the changes in the current with respect to the voltage. The silicon nanowires were treated as resistors, being placed in FETs with a Schottky source and drain contacts. The sensors were tested with three antigen concentrations, which showed a rise in the capacitance with respect to the voltage gap. The concentrations of antigen varied between 0 and ten fM. The gap was created due to the occurrence of the functionalization of the antigen with the antibodies. This change occurred due to the change in the Schottky barrier as a result of the change in the electric potential during the injection of the carriers. This study proved that the behaviour of the physical system was in accordance with the bio-modified surface as a result of the changes in the capacitance of the nanowires.

The testing of these nanowire-based memristive devices was also done for the prostate-specific antigen (PSA) IgM detection. A label-free detection of the biomarker was performed, where three low concentrations of the PSA were tested and analysed. These experiments were conducted to validate the performance of these devices for the early detection of prostate cancer disease. The results were cross-checked with an enzyme-linked immunosorbent assay (ELISA) technique to further determine their accuracy. The substrates of the silicon nanowires were functionalised with different antibody solutions via an over-night incubation. The concentration of the PSA-IgM was varied between 11.75 and 47 AU/mL, with the precision being maintained by removing the excess of the solution using 1.5 mL of PBS-Tween 0.05% and MilliQ water. The drain to source voltage was swept between −2.4 V and 2.4 V to determine the changes in the current under an environment with a controlled current and humidity. A hysteretic loop was obtained for the current-to-voltage characteristics, with a voltage gap being created after the bio-modification of the nanowires. The charged residues of the proteins changed the characteristics of the electric field, which subsequently changed the conductance of the device.

### 2.7. Piezoresistive Sensors

The design of each of the silicon-based sensors varied with the type of application they were being used for. In addition to the traditional piezoresistive sensors developed by different polymeric materials, silicon-based piezoresistive sensors have served a better purpose in this respect as they have been advantageous for a wide range of pressure-based biomedical applications. Some of the advantages of these sensors are their improved accuracy, expanded pressure range, lower power consumption and reduced cost of production. Beccai et al. [87] explained the development of a hybrid three-axial sensor to force sensing in biomechanical applications. The sensors were developed on a 525 µm wafer having a high aspect-ratio cross-shaped flexible element. The piezoresistive working principle was followed by these sensors, with four resistors operating in a combined fashion, each one having a range of length and width of 30–50 µm and 6–10 µm, respectively. A hybridised integrative approach was adopted for the interconnection and operation of these force sensors to achieve a high linearity and low hysteresis for both normal and shear forces. The sensor was designed to have a combined connection of carrier chip and flip-chip technology. The final sensor had dimensions of 2.3 mm × 2.3 mm × 1.3 mm. The sensors were used to determine the change in the relative resistance concerning the different forces applied to them.

One of the studies highlighting the use of silicon-based sensors for neural studies was done by Manikandan et al. [88] by designing and developing intracranial pressure sensors. The design of these sensors was based on the multi-electrode array, where the size of each sensor was dependent on the size of the chip and package. The size of the total chip was around 100 × 200 µm^2^, where each of the sensors operated based on a piezoresistive effect. The total sensing chip also consisted of a temperature and a separate pressure sensor. The resistance measured from the sensor array was based on the Wheatstone bridge to operate the sensors as passive resistive resonant prototypes, in order to accurately determine changes with respect to intracranial pressure. The sensitivity of the system was around 0.84 × 10^−2^ mV/kPa for a maximum pressure limit of 112 Pa. The sensors consisted of four round-shaped microelectrodes developed from Silver and Platinum. The entire array was divided into three sides, consisting of simulation sites, electro-physiological sites, and a pressure sensing side. There were some other sites that were kept open in the sensor array for the detection of certain proteins like glutamate oxide, from a matrix layer. The connecting pads for each of the two electrodes had a size of around 500 µm × 500 µm for the transfer of the monitored signals.

The two electrodes were used as counter and reference parts, where the difference in the voltage signals between them was measured by the embedded detecting circuit. The fabrication of the sensor was done on silicon substrates, with a thin layer of polyimide having a thickness of 15 µm being coated to form the base for the electrodes and temperature sensor. After the electrodes were formed using Au and Pt, another polyimide layer was coated on the surface. This was followed by the defining of the electrodes by performing a reactive ion etching technique on the top layer to form the openings. The resistive temperature sensors were formed by sputtering Silver under temperature and vacuum conditions of 350 °C and 1 × 10^−6^ mbar, respectively. The electrodes were positioned at specific locations to determine the pressure of the neurons without the assistance of any special gel. In the presence of the four sensors, two of them were calibrated to measure the variation of the pressure. The measurement of the temperature was done between the range of 0 and 50 °C to calibrate the sensor and determine the linearity of their responses. The sensing chip was able to respond between the range of −20 °C and 20 °C, having an accuracy of around 89% in the response. The effect of the external pressure on the piezoresistive sensors was also determined with the change in resistance.

One of the earlier works related to the use of silicon-based piezoresistive sensing was done by designing and developing pressure sensors for biomedical applications [89]. The sensors were fabricated to respond to low pressure to achieve an enhanced performance factor that was defined by the product of the signal-to-ratio, sensor sensitivity and temperature coefficient of the piezoresistance. The sensors achieved the highest figure of merit for the sensors having a dimension and thickness of 100 µm and 10 µm, respectively. The optimisation was done based on a mathematical calculation to determine the output voltage of the sensor, sensitivity, noise, and signal-to-noise ratio. The doping concentration of the sensors was inversely proportional to their sensitivity and directly proportional to the signal to noise ratio. The coefficient of the temperature initially increased with the doping concentration and then decreased after reaching a certain value. The optimised values of the thickness of the diaphragm, doping concentration, and length of the sensor were 15 µm, 5 × 10^17^ atoms cc^−1^ and 25 µm, respectively.

Despite the reduced size of these sensors, they could also respond to a varied range of temperatures at a high sensitivity and pressure limit. The fabrication process of the sensors is also compatible, as the sensing area was developed using standard MEMS-based techniques. One of the factors that is instrumental in determining the performances of these sensors is the doping level, which significantly varies the response to a range of pressures, thus affecting its sensitivity.

### 2.8. Integrated/Hybrid Sensors

The last category of silicon-based sensors is based on the formation of different kinds of sensing elements for multifunctional biomedical applications. These sensors are different from other types, as explained above in terms of the fabrication technique and the resultant prototypes. Another significant attribute of these types of sensors is their customised nature, which increases the range of applications they can be employed for. Instead of following a specific working principle specialised in the application type, integrated sensors were found to operate on more than one kind of principle. One study explains the silicon-based blood pressure sensor designed by Wu et al. [90] using the fractional flow reserve (FFR) technique. The diameter of the sensor is 125 μm, and a reference manometer was used to compare the pressure results. Due to the examining capability of the location of a stenosis in the coronary artery, the fibre optics have a great advantage in the fabrication of this sensor. The use of a silicon dioxide diaphragm of uniform thickness eliminates the bulky structure of the sensor head. During the blood pressure measurement, the inflation/deflation cycle gives an electric voltage signal from which the results of each transient period are received. First, the sensor receives information regarding the heartbeat signal, and then the blood pressure is monitored at different positions in the coronary artery. Satake et al. [91] fabricated a micro silicon-based sensor that can count the number of blood cells in human blood and the latex particles using the Coulter method. It is also possible to distinguish the white and red blood cells via this microsensor. A glass cover attached to a silicon substrate was used to develop this particular sensor, which has a dimension of 10 mm × 5 mm × 0.8 mm. The researchers proved that the presence of linearity between red blood cells and the counts could be measured. The height and the number of pulses help to determine the differences in size and the concentration of PSL particles, respectively. One of the advantageous attributes of these sensors is that they can be fabricated using both glass and silicon. The smaller dimension of the sensors is another advantage to it.

A multifunctional silicon-based sensor was designed by Kang et al. [92]; it was tested by being implanting in the brain of a rat. This bio-resorbable electronic sensor has the wireless ability to monitor the intracranial pressure (ICP) and intracranial temperature (ICT) regularly and simultaneously, which can be very helpful for the treatment of a traumatic injury of the brain. This research group utilised a wireless transmitter-based sensor for the data transmission. The fluid flow and motion, thermal characteristics and pH can also be obtained, which are related to sudden pain in the body when they are installed in deep tissues or body cavities.

**Figure 4:**
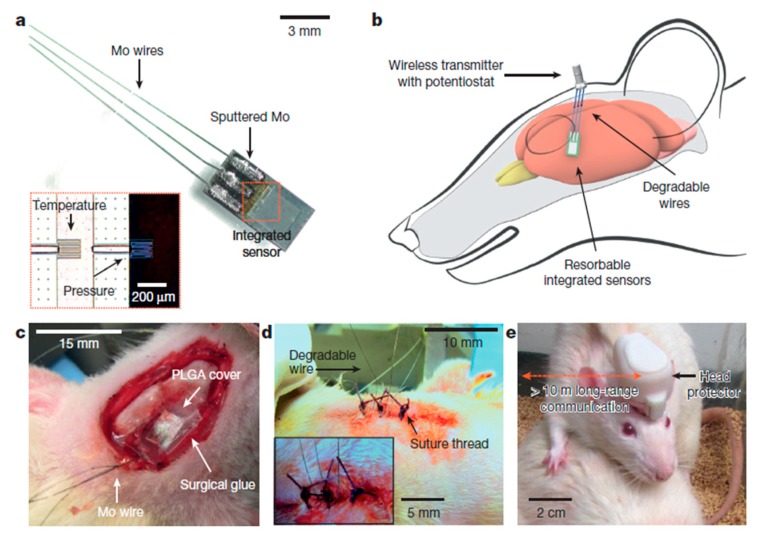
Schematic diagram to represent the interfacing of Bioresorbable sensors with the communication modules for the wireless data transfer. The image also shows the connection of the bioresorbable pressure and temperature sensors integrated with the dissolvable metal interconnect [92]. The image has been reproduced with permission from [92].

This biosensor is not only helpful for the diagnosis of chronic health illnesses such as diabetes, but also very helpful for the treatment of the cardiac space and spinal system by sensing and stimulating. Figure 4 depicts the schematic diagram of the location, on the rat, of the sensors for pressure and temperature sensing using wireless communication [92]. The key characteristics of this sensor include the measurement of multiple intrinsic parameters of the brain, along with the determination of its behaviour for other physio-chemical features.

Hui et al. [93] have showcased work related to the use of silicon wafers to develop bio-integrated flexible electronic systems. The integration of silicon wafers was done with flexible electronics, the key characteristic of these sensors. The integration of two types of materials led to the achievement of the enhanced electrical conductivity and mechanical flexibility of the sensors. The thickness of the layers grown on the wafers was under 1 µm. The sensors have been proven to be effectively used for chronic implants. Thermal oxidation on silicon wafers was done to grow a thin layer of silicon dioxide; the process was followed by electron beam evaporation, photolithography, and etching to achieve specific electrode patterns. Magnesium was encapsulated by two layers of silicon dioxide and a mixed layer of aluminium oxide and Parylene C. The layer of magnesium with a thickness of 200 nm was formed to examine the behaviour of the sensors toward the water barrier properties. The silicon dioxide layers were then transferred to a thin polyimide film having a thickness of 25 µm. The polyimide film was supported by a temporary glass substrate for manual manipulation purposes. Electrochemical impedance spectroscopy (EIS) was used to detect the performance of the thermal growth of the silicon dioxide layer by developing an equivalent circuit. The circuit consisted of two capacitive elements, namely oxide and double-layer capacitances, and two resistive elements, solution and charge-transfer resistances. Although the testing of these hybrid sensors was done using one element, the response of these sensors toward EIS has opened a huge potential in determining the response of these types of sensors for other analytes, such as proteins, for a range of frequencies. The electrochemical cell responding to the frequencies can be optimised by including different kinds of polymers to conjugate with silicon and other metallic elements.

One of the studies explaining the use of silicon carbide for biomedical applications has been showcased by Gabriel et al. [94] in the form of multi-sensor multi-probes. The use of silicon carbide as a semiconducting substrate has been popular for the last decade due to its capability of working in high-power applications. It was seen that the performance of silicon carbide as a substrate was better than that of silicon from the particular set of experiments that were performed to determine the change in impedance with respect to the frequency for different tissue properties. Initially, a layer of Pt/Ti with a thickness of 450 µm was deposited on silicon and silicon carbide, where the former substrate was oxidised by CVD to form an SiO_2_ layer of 1.5 µm. The area of the system was around 14.85 × 0.83 mm^2^ with the presence of two pair of electrodes, each one having an area of 300 × 300 µm^2^. The sensors were mounted on printed circuit boards with gold contacts, followed by the electrodes being platinised. This was done to reduce the total impedance of the sensors. The treatment was done with KCl solutions, along with platinum chloride and lead acetate. The in-vitro measurements were done with a four-electrode sensor via the immersion of the impedance probes in the diluted and non-diluted physiological solution. The frequency sweep was done between 10 Hz to 1 MHz to determine the changes in the impedance and phase angle for both the silicon and silicon carbide substrates. It was found that the silicon carbide substrates outperformed the silicon sensors in terms of the load-deflection tests, in-vitro characterisations, and deflections, and this simulated life-like situations. The importance of silicon carbide operating on a range of frequency sweep can be used to exploit its performance for other in-vitro and in-vivo applications. The use of silicon both as electrodes and substrates does create a uniform structural attribute in terms of the behavioural aspect. These sensors were also smaller in size, which makes them suitable for determining the changes in some of the elements inside the body at minuscule levels.

**Figure 5:**
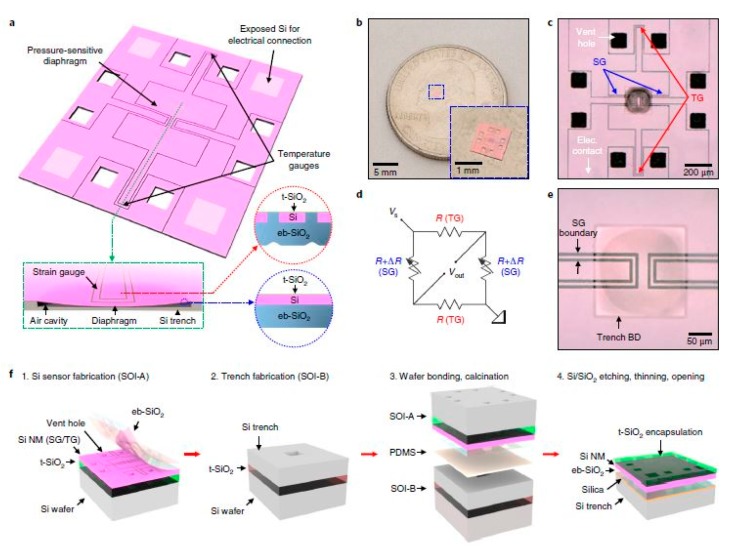
Schematic diagram of the fabrication process of the bioresorbable pressure sensors. The sensors were developed with monocrystalline silicon and silicon dioxide layers using thermal oxidation and electron-beam techniques. Two boundary lines were used to separate the strain gauges from the surrounding silicon. The system consisted of piezoresistive sensors, a voltage source and a meter to compensate for the variation of the temperature caused by the variation in the resistance with the pressure [95]. The image has been reproduced with permission from [95].

One of the recent silicon-based works for medical applications has involved the development of bioresorbable pressure sensors for chronic diseases and healing processes [95]. Some of the diseases being dealt with by these pressure sensors are traumatic brain injuries, glaucoma and hypertension, based on the pressure in different parts, like the intracranial, intraocular and intravascular spaces, respectively. The experiments were conducted on rats for 25 days to validate the lifetime and repeatability of the response of the sensors. Apart from the sensors being biodegradable, the results in terms of the processed materials, blood counts, blood chemistry, and magnetic resonance imaging compatibility showcased the clinical usage of the device. Figure 5 shows the schematic diagram of the fabrication steps of the sensors [95]. The sensing system consists of four silicon nanomembranes, each having a thickness of 200 nm. The diaphragm of the system was developed to form a floating, pressure-sensitive region of air-filled cavity, having the dimensions of 200 µm × 200 µm × 10 µm. The temperature sensor was located away from the diaphragm, so that the response of this sensor would not get affected by the pressure. The diaphragm consisted of monocrystalline silicon and evaporated silicon dioxide layers of 200 nm and 600 nm, respectively. The electrode layers were developed with silicon nanowires, with overall dimensions of 200 µm × 200 µm. The sensing system was operated as a Wheatstone bridge consisting of a voltage source, temperature sensor and four pressure sensors. The thickness of the silicon nanowires was optimised to increase the pressure sensitivity of the device. The resistance values of the sensors changed with respect to the temperature in order to obtain a linear response, having a temperature coefficient of 0.0012 °C^−1^. The sensitivity of the pressure sensors varied within a range of ± 1.5% for 22 days. The sensors were mounted on a thin film of poly (lactic-co-glycolic acid) and bonded with the surrounding skull with glue. The outputs of the sensors were measured in terms of the change in voltage with respect to the time for different structural configurations of the sensors. Apart from their bioresorbable nature, some of the biggest advantages of these sensors lie in their small size, their capability of performing precise measurements in delicate parts of the body, their consistency in the results, and the very low dependence of their responses on the change in temperature.

## 3. Current Challenges and Future Opportunities

Although a lot of work has been done in relation to silicon-based biomedical sensing, there are still some existing challenges that need attention in the current scenario. Starting from the fabrication of single-crystal silicon for forming wafers, it is an expensive process and requires highly-equipped refineries. In today’s world, these refineries cost more than three billion dollars [96]. This is the reason why there is not much change in the cost of a single silicon chip. Second, in comparison to other substrates, silicon-based sensors, if produced in a lower quantity, would cost more per unit, unless ordered in bulk. Furthermore, toxic by-products are produced during the fabrication of silicon wafers [97]. This reduces their chances of being considered as useful for intrinsic biomedical applications. In comparison to the currently available organic conductive materials that have a high aspect ratio, silicon has a smaller sensing surface area, which eventually reduces its performance in terms of sensitivity and efficiency. The increase in the surface area would require an increase in the total size of the sensor, thus increasing the cost of the sensor per unit. Third, the thin-film wafers are very brittle, thus making it difficult to integrate them with signal-conditioning circuits in a more cost-efficient way. Another major disadvantage of the silicon-based sensors is the dependence of their responses on the change in temperature. Even though the sensors do perform well within the range of the temperature of the human body, these sensors possess a limitation when used for monitoring anatomical changes inside the human body. Additionally, due to the semiconducting nature of silicon, it is one of the least feasible options for forming the electrodes with the presence of highly conductive materials like gold, aluminium and others. One of the major factors that is limiting the use of silicon sensors for biomedical sensing in the current scenario is the limitation of its biocompatibility in comparison to the currently available carbon compounds. The biocompatibility should be increased via integrating or doping the silicon sensors to make them further suitable for implantable applications. Their response toward the analyte molecule also needs to be increased to decrease the interference of other elements during the experimental process. This is particularly necessary in the case of in-vivo applications, where the anatomical system of human beings and other animals is very complex. The inclusion of the presence of other bio-analyte, even in a very small amount on the sensing surface, can result in erroneous results. Other disadvantages of silicon-based sensors include their irregular behaviour and high signal-to-noise ratio (SNR) at a low frequency. This creates other problems at a higher frequency, such as a high input power and inconsistency in the data.

One of the remedial measures that can be considered to tackle the challenges mentioned above is the amalgamation of silicon with other materials to form both the substrates and electrodes of the sensors. For example, researchers have been making nanomaterials like silicon carbide on a large scale to form the electrodes for biomedical sensing [98]. The electrical and thermal characteristics of these nano-compounds can be affected by including certain elements like nitrogen and aluminium as impurities during the fabrication process. Similar to the conductive parts, silicon substrates would also be advantageous, especially for biomedical sensing. The flexibility of the substrates would allow for interfacing with certain intricate organs like the brain, in order to determine the signals from the soft tissues [99]. In terms of fabrication, doped-silicon electrodes can be formed on the prototypes by using soft lithographic techniques. Silicon can also be used as the master mould to form the designated electrode designs. This would be advantageous in terms of cost-operating conditions. Certain standard polymers like PDMS can be used as the stamp to form the final prototypes at room temperature. Furthermore, techniques like soft lithography can assist in quickly and efficiently obtaining 3D structures. The premise of using doped-silicon, along with silicon substrates, would also minimise the probability of affecting the characteristics of the final prototypes. This technique has a potential that would prove it to be better than the conventional photolithographic technique in terms of its resistance to the diffraction of light. Another primary advantage of using this technique is the possibility of the development of new 3D structures for biomedical applications that cannot be easily obtained by commonly used techniques. Researchers have been working [100,101] on this technique in conjugation with softer materials like organic polymers to obtain hybrid materials for healthcare applications. The sensors developed with this method have also performed with a high robustness, high efficiency, and enhanced mechanical and thermal characteristics [102]. Another solution for silicon-based sensors related to the high SNR at lower frequencies can be the use of biodegradable [103] and non-biodegradable [104] silicon nanowires for monitoring purposes. An alteration of the process of fabrication of silicon wafers can also be implemented to reduce the production of harmful by-products. This would also have an influence on their uses for biomedical sensing, as optimised fabrication processes would generate wafers with a better biocompatibility. One of the techniques for doing this is the fabrication of compound semiconducting wafers, which include materials such as Gallium Nitride, whose properties are similar to silicon. To reduce the SNR of silicon wafers, their sizes can be reduced to below 300 mm [105], which will not only allow an amplified signal for a high range of frequencies, but will also allow the reduction of the required power to drive the sensing systems. The application of the prototypes for biomedical sensing can be increased by making the silicon sensors multi-functional, operating on different genres. For example, an array of sensors can be formed to induce both electrochemical and strain-sensing purposes with the same system. This will reduce the total cost of the system while simultaneously improving their dynamic nature and portability. The range of parameters in biomedical sensing that are being monitored and diagnosed by the current sensors can be increased by including selectivity for specific applications. Sensors should be developed where the electrodes are intermixed with the template molecule for a particular analyte. In this way, the process of separately coating the sensing surface of the sensors can be eliminated.

The market surveys done on silicon-based sensors predict growth in their usage for biomedical sensing in the next few years. It has been estimated that the use of silicon would be extensively implemented to develop different kinds of medical devices, including pharmaceuticals, implants, and others [106]. The use of sensors in the form of microfluidic devices has been estimated to rise in fifteen years [107]. These silicon-based devices have been mostly used for pharmaceuticals, drug delivery, and in-vitro analyses. The market for silicon-based microfluidic devices was seen to be around 10.2 billion USD by 2017 and is predicted to rise in the future. The silicon sensors would also be used in the form of silicon carbide to make sensors for health care operations. It has been estimated that the rise in the use of silicon carbide materials would increase more than 1 billion USD by 2025, with a compound annual growth rate of 18.15% [108]. This increase in the cost of silicon sensors could indicate a rise in their subsequent uses in developing medical devices of a varied type. It is thus anticipated that there will be a prominent use of silicon-based sensing technology in the biomedical field for the ubiquitous monitoring of different acute and chronic diseases.

## 4. Conclusions

This paper explains the significance of the employment of silicon-based sensors for biomedical applications in the last two decades. Some of the important research in this area has been highlighted to define the fabrication and utilisation of different kinds of silicon-based prototypes for biomedical sensing purposes. The use of silicon to develop sensors has been advantageous in terms of their high abundance, low SNR, high thermal stability, resistance toward response changes based on ambiance conditions, high sensitivity and reliability, high repeatability in their response, low variation of response with time, low response time, and huge prosperity in terms of the future market. Apart from this, the electrical conductivity and mechanical flexibility of silicon have varied because of different methods, like the doping of elements to use them for varied purposes. Right from the fabrication of semiconducting substrates in the conventional MEMS technology, they have also been used to form nanowires in the prototype of the sensor. The above sensors have shown responses to in-vitro and in-vivo experiments for electrochemical analyte from animals and human beings. The use of silicon sensors for multifunctional applications is something that needs to be targeted in the future. This will not only minimise the cost of fabrication of the sensing systems but will also reduce the amount of generated electronic waste. The fundamental characteristics of silicon need to be further altered to increase their dynamicity in terms of the types of fabricated prototypes. The miniaturisation of sensors in the current world is one area where silicon-based sensors need to be fitted to utilise their advantages, as mentioned above. The conjugation of silicon with other conducting and semiconducting elements will assist in developing nanoparticle-based prototypes to further determine the minuscule intrinsic changes taking place in the body. The behaviour of these integrated sensors could, to a large extent, dictate their quality in the monitoring of biomedical applications.

## Figures and Tables

**Table 1:** A comparative study of the different silicon-based sensors used for biomedical sensing.

Materials	Technique of Fabrication	Application	Strengths	Ref.
Silicon-based MEMS Electric Condenser Microphone	Semiconducting production processing	Human pulse detection	Smaller size, better quality than other ECMs	[21]
Silicon Nanowire	Bottom-up approach	Detection of DNA molecules	Thermally and chemically stable, interconnects better with the components	[35]
Silicon probe, PEDOT: PSS, polyimide	Monolithic microfabrication process	Detection of neural activity	Implants several probes in the brain within a short time	[36]
Silicon-based CMOS and BiCMOS	Photolithography and chemical process	Heartbeat and respiration activity	Wireless communication, high data transfer rate	[37]
Amorphous silicon-image sensor based on thin-film transistors	Thin-film semiconducting process	X-ray Diagnostic Medical Imaging	Low data lines capacitance, noise cancellation techniques and optimised timing	[38]
Silicon-based CMOS and BiCMOS	Photolithography and chemical process	Detection of peripheral and cranial nerve activities	Enhanced biological and electrical performance of the implantable sensors	[39]
Silicon-Silicon dioxide-Chromium	Conventional photolithography process	Detection of proteins and photo lipids	Reduced electrode impedance, higher sensitivity, reduced dependence on cell mobility	[40]
Nitrogen-doped silicon	Thermal oxidation and deposition	Detection of protein (Avdin)	Low detection limit and high sensitivity	[41]
